# Reviewers in 2024

**DOI:** 10.1098/rsob.250082

**Published:** 2025-05-07

**Authors:** Jon Pines

**Affiliations:** ^1^ Editor-in-Chief

**Figure:**
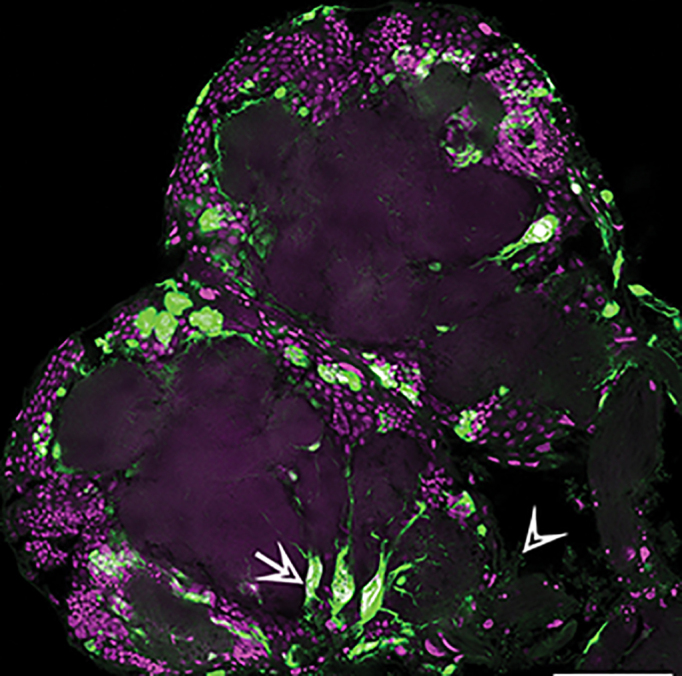

The Editors of *Open Biology* would like to thank all reviewers who contributed their time by refereeing manuscripts submitted throughout 2024. From previous feedback, we know that most reviewers value recognition of their efforts by the journal and through services, such as Web of Science Reviewer Recognition Science, which is integrated with *Open Biology*.

Under our scheme to reward peer review (https://royalsocietypublishing.org/rsob/reviewer-rewards), reviewers of *Open Biology* are awarded tokens that can be redeemed against article processing charges (APCs) when they publish with us.

To further provide opportunities for recognition, we list the names of all reviewers who opted in. This article is made permanent and citeable by its digital object identifier (DOI). We encourage you to quote your contribution in your applications for tenure and grants or other forms of research assessment. Where you have provided it, we have included your ORCID, so your contribution can be unambiguously assigned to you.

The team really does appreciate all the work our reviewers do on behalf of the journal, and we look forward to working with you again in future.

Abdel-Daim M

https://orcid.org/0000-0002-4341-2713

Akan O

Aksnes H

Alfieri C

Archambault V

https://orcid.org/0000-0002-2857-7667

Ardern Z

Awosile B

Azzalin C

Barral DC

Barrick J

Bartelt A

Battersby BJ

Becker T

Behr R

Bendotti C

Beneke T

Berndt S

Bernués J

Bessho-Uehara M

Beukeboom L

Birge R

Blackburn D

Brill J

Broughton SJ

Budzak J

https://orcid.org/0000-0001-9336-6789

Cai L

https://orcid.org/0000-0001-5976-2382

Carling D

Castro LF

https://orcid.org/0000-0001-7697-386X

Chan F

Chen S

https://orcid.org/0000-0003-4565-4772

Chirinos M

https://orcid.org/0000-0003-0023-5195

Clayton C

https://orcid.org/0000-0002-6384-0731

Clement J

https://orcid.org/0000-0001-7625-6430

Czogalla A

https://orcid.org/0000-0003-3035-059X

Dabrowska J

Dallai R

Dan S

de Graffenried C

https://orcid.org/0000-0003-3386-6487

de la Serna IL

de Pereda JM

https://orcid.org/0000-0002-8912-6739

De Stefani D

Del Olmo N

Derat E

Diallinas G

https://orcid.org/0000-0002-3426-726X

Dolezal P

https://orcid.org/0000-0003-1285-9026

Drin G

Eme L

Escriva H

Fairweather SJ

Farraj PA

Fedosova N

Fehl C

https://orcid.org/0000-0001-6182-0879

Florent I

Fontanesi L

Forsdyke D

Fransen M

Freitak D

https://orcid.org/0000-0001-8574-0531

Funk M

https://orcid.org/0000-0002-6007-1164

Galati D

Galizia CG

https://orcid.org/0000-0001-8292-6031

Garcia Macia M

Gendrin M

Georgel P

Giet R

Giglia-Mari G

Gilberger T-W

Gillen A

Gomes E

Gontijo N

Green II D

https://orcid.org/0000-0002-3373-8867

Gupta N

Gómez-Moracho T

https://orcid.org/0000-0002-3063-8617

Halberg KA

Hayashi D

Helfrich-Förster C

https://orcid.org/0000-0002-0859-9092

Herrmann H

Herskind C

https://orcid.org/0000-0001-6554-5907

Higgins J

https://orcid.org/0000-0001-6622-8268

Hirata Y

Hong Y

https://orcid.org/0000-0002-4408-2648

Hrcek J

https://orcid.org/0000-0003-0711-6447

Hundry B

https://orcid.org/0000-0003-1299-843X

Ibarra LE

Irwin N

Jiao R

Joanna C

https://orcid.org/0000-0001-7613-8127

Jones C

https://orcid.org/0000-0002-6504-6224

Jonsson H

Jontes J

Kamikawa R

Knippschild U

Kornblihtt A

Krappmann S

Krueger A

Kruit JK

Kunz L

https://orcid.org/0000-0003-3141-0005

Kuscu M

Lamming D

Larhammar D

Laurentino S

Li C

Li C

https://orcid.org/0000-0002-1950-0190

Li Y

Lin S

Lopez LM

Lu K

Madrid M

Maiga H

Maione S

Mannion A

McKean P

Medigeshi GR

Megraw T

Mizotani Y

Moberg K

https://orcid.org/0000-0002-0859-9092

Mondal P

Monteiro R

Mukherjee AK

Murakami M

Musacchio A

Naeem M

Nakagawa S

Nawy S

Nemazee D

Nikalayevich E

Nisbet E

Ochsenreiter T

https://orcid.org/0000-0002-8846-8526

Pabst G

Paillat M

Panich U

https://orcid.org/0000-0002-4889-6729

Pantazis A

https://orcid.org/0000-0002-6467-1327

Parikh AN

Pazoki Toroudi HR

Perry T

Polevoda B

Poulin LF

Putkey J

Rajan N

Raju Sunil KK

Ranea Robles P

Rauch I

Regensburger M

Rodriguez-Casariego JA

https://orcid.org/0000-0001-9955-4721

Romanova D

Ruelland E

Sachkova MY

Sahoo D

Samways D

Schiebel E

https://orcid.org/0000-0002-3683-247X

Serna IL de la

Sharma S

Shu-Chien AC

Silver A

Smith-Bolton RK

Soroka M

Strong T

Sudyka J

https://orcid.org/0000-0003-3006-3806

Sumanas S

Susor A

Swaminathan VS

https://orcid.org/0000-0002-9946-810X

Takaori A

https://orcid.org/0000-0001-7678-4284

Tian X

https://orcid.org/0000-0002-5601-2057

Tomášek O

https://orcid.org/0000-0002-2657-5916

Tropepe V

Tsurumi A

Valenzuela R

Valenzuela Sánchez A

van Wijnen A

Varela S

Verma DP

Viney M

Wade J

Wakarchuk W

Wake M

Wang H

Wang JWJL

Wehman AM

https://orcid.org/0000-0001-9826-4132

Wideman J

Wollscheid H-P

Yang D-L

Yang J

Yang Z

Yano D

https://orcid.org/0000-0003-2672-3525

Yvinec R

Zang C

Zapotoczna M

Zelena D

Zhang G

Zhang J

Zhang X

Zhou Z

